# Turn Performance Variation in European Elite Short-Course Swimmers

**DOI:** 10.3390/ijerph19095033

**Published:** 2022-04-21

**Authors:** Francisco Cuenca-Fernández, Jesús J. Ruiz-Navarro, Marek Polach, Raúl Arellano, Dennis-Peter Born

**Affiliations:** 1 Aquatics Lab, Department of Physical Education and Sports, Faculty of Sport Sciences, University of Granada, 18011 Granada, Spain; cuenca@ugr.es (F.C.-F.); jesusruiz@ugr.es (J.J.R.-N.); r.arellano@ugr.es (R.A.); 2 Department of Social Sciences in Kinanthropology, Palacký University Olomouc, 77147 Olomouc, Czech Republic; marek.polach01@upol.cz; 3 Department for Competitive Swimming, Czech Swimming Federation, 16017 Prague, Czech Republic; 4 Section for High-Performance Sports, Swiss Swimming Federation, 3063 Bern, Switzerland; 5 Department for Elite Sport, Swiss Federal Institute of Sport Magglingen, 2532 Magglingen, Switzerland

**Keywords:** freestyle, competition analysis, performance, race analysis, swimming

## Abstract

Turn performances are important success factors for short-course races, and more consistent turn times may distinguish between higher and lower-ranked swimmers. Therefore, this study aimed to determine coefficients of variation (CV) and performance progressions (∆%) of turn performances. The eight finalists and eight fastest swimmers from the heats that did not qualify for the semi-finals, i.e., from 17th to 24th place, of the 100, 200, 400, and 800 (females only)/1500 m (males only) freestyle events at the 2019 European Short Course Championships were included, resulting in a total of 64 male (finalists: age: 22.3 ± 2.6, FINA points: 914 ± 31 vs. heats: age: 21.5 ± 3.1, FINA points: 838 ± 74.9) and 64 female swimmers (finalists: age: 22.9 ± 4.8, FINA points: 904 ± 24.5 vs. heats: age: 20.1 ± 3.6, FINA points: 800 ± 48). A linear mixed model was used to compare inter- and intra-individual performance variation. Interactions between CVs, ∆%, and mean values were analyzed using a two-way analysis of variance (ANOVA). The results showed impaired turn performances as the races progressed. Finalists showed faster turn section times than the eight fastest non-qualified swimmers from the heats (*p* < 0.001). Additionally, turn section times were faster for short-, i.e., 100 and 200 m, than middle- and long-distance races, i.e., 400 to 1500 m races (*p* < 0.001). Regarding variation in turn performance, finalists showed lower CVs and ∆% for all turn section times (0.74% and 1.49%) compared to non-qualified swimmers (0.91% and 1.90%, respectively). Similarly, long-distance events, i.e., 800/1500 m, showed lower mean CVs and higher mean ∆% (0.69% and 1.93%) than short-distance, i.e., 100 m events (0.93% and 1.39%, respectively). Regarding turn sections, the largest CV and ∆% were found 5 m before wall contact (0.70% and 1.45%) with lower CV and more consistent turn section times 5 m after wall contact (0.42% and 0.54%). Non-qualified swimmers should aim to match the superior turn performances and faster times of finalists in all turn sections. Both finalists and non-qualified swimmers should pay particular attention to maintaining high velocities when approaching the wall as the race progresses.

## 1. Introduction

In highly technical sports, such as swimming, overpacing at the beginning of the race may dramatically interfere with swimming technique towards the end, hence impairing movement efficiency [1]. Faster athletes showed more even pacing, with a slower start but less time lost towards the end of the race [2,3]. Medalists at major swimming championships even conserved enough energy to accelerate during the last lap of the race, thus producing a more pronounced end-spurt than their lower-ranked competitors [4]. Furthermore, as less experienced swimmers, i.e., juniors, showed higher variation in their pacing pattern [2], well-adjusted and highly reliable pacing might be an important success factor during high-profile swim races.

The coefficient of variation (CV) has typically been used to investigate the intra-individual variation in the performance of short-, middle-, and long-distance swimming events [5,6,7,8,9]. Any change in performance showing a CV ≥ 0.5% is considered relevant for practice [5]. However, variations may differ across the different sections of swim races, i.e., start, turn, clean swimming, and finish [10,11]. Indeed, in addition to interfering with clean swimming performance, the aforementioned positive pacing pattern also affected turn performance towards the end of a 200 m race simulation [12]. Race analyses confirmed this effect in finalists of the 2019 European Junior Championships showing significantly slower turn times for the last than the first turn of the race [13]. However, turns are typically split into turn section times before and after wall contact [10]. Regarding these turn section times, variation was largest 5 m before wall contact [13,14] and similar to the variation in clean swimming performance [13]. However, there was a lower variation for the first 15 m after wall contact [13], probably due to the streamlined body position adopted during the underwater phase, making this section less fatigued. Although velocity is well maintained in one of the multiple turn sections, fatigue or changes in race strategy in another turn section of more than the 0.5%, as mentioned earlier, may result in a significant performance deterioration over the total race time [15]. Therefore, variation of turn performances must be analyzed individually and sequentially for each turn section.

Variation and pacing patterns in turn performance may be particularly important success factors regarding short-course races (25 m pool length) due to the high contribution of turn performances (up to 56.08 ± 0.28% in 1500 m freestyle) to race time [16]. The push-off from the pool wall doubles movement velocities after turns (2.96 ± 0.14 m/s) compared to the clean swimming (1.41 ± 0.06 m/s) sections [17]. Thus, the increased number of turns in short-course races increases average race velocity by 2.0 ± 0.6% compared to long-course races [18], resulting in a close correlation between the initial 10 m following wall contact and total race time (*r* = 0.83) [17]. A new perspective on turn performance on development in the 1500 m freestyle performance was demonstrated by a detailed analysis of the 2018 and 2019 world championship events [19]. On average, swimmers turned 0.07 s faster in long- compared to short-course races. The greater number of turns in short-course races performed at this faster pace indicates the potential to improve the world record by 2.10 s [19]. Indeed, despite slightly slower clean swimming times, the world record was recently improved by 1.18 s in the men’s 1500 m freestyle event at the 2021 Short-Course World Championships, which was attributed to the overall 2.30 s faster turns [20].

As turn performances provide important success factors for short-course races [16,19,20] and become progressively slower throughout a race [12,13,19,21], more consistent turn times may distinguish higher- from lower-ranked swimmers. Furthermore, pacing strategies differ between short- and long-distance races [2,6,7,13,14,21]. As such, variation and consistency in turn section times may be affected by the distance of the race. Therefore, the study aimed to investigate the effect of variations in short-course turn performance in elite swimmers at the European Short-Course Championships. The research questions were whether variations in turn performances differ between (1) the various turn sections, (2) race distances, and (3) performance levels, i.e., finalists vs. non-qualified swimmers.

## 2. Materials and Methods

### 2.1. Subject Characteristics

The present investigation analyzed the turn performances of 128 races from the 2019 European Short-Course Championship. The eight finalists (Performance Level 1) and eight fastest swimmers from the heats that did not qualify for the semi-finals, i.e., from 17th to 24th place (Performance Level 2 [22]) of the 100, 200, 400, and 800 (females only)/1500 m (males only) freestyle events were included in the study, resulting in a total of 64 male (finalists: age: 22.3 ± 2.6, FINA points: 914 ± 31 vs. heats: age: 21.5 ± 3.1, FINA points: 838 ± 74.9) and 64 female swimmers (finalists: age: 22.9 ± 4.8, FINA points: 904 ± 24.5 vs. heats: age: 20.1 ± 3.6, FINA points: 800 ± 48). As 800 m for males and 1500 m for females were introduced to the Olympic freestyle events in 2021, these events were not included in the present study [23]. The study was approved by the Institutional Review Board (IRB) of the Swiss Federal Institute of Sport Magglingen (Reg.-Nr. 098-LSP-191119) as part of a larger investigation of swimming race data [16,24] and is in accordance with the ethical principles for studies involving human subjects (Helsinki Declaration) of the World Medical Association (WMA). No written informed consent was required as participants of the European Swimming Championships are video monitored for television broadcasting and race analysis by the organizer of the event Ligue Européenne de Natation (LEN).

### 2.2. Data Collection

The swim races were video monitored using a 12-camera system (Spiideo, Malmö, Sweden). Ten moving view cameras (V59 PTZ, Axis Communications AB, Lund, Sweden) followed the swimmers on each lane individually. Two fixed cameras were positioned at each end of the pool at a 90° angle and monitored the starts and turns across all lanes from the side. All cameras were synchronized and collected video footage at 50 Hz. Final standings and race times for each event were provided by the official timekeeper of the championships (Microplus Informatica, Marene, Italy). Subsequently, video footage was digitized manually using video analysis software (Kinovea 0.9.1; Joan Charmant & Contrib., kinovea.org, accessed on 10 August 2020). Markings at the lane ropes were used to identify when the top of the swimmer’s head passed 5 m before and 5 m and 10 m after the pool wall. Contact of the feet with the wall following the rotation of the flip turn determined the end of each lap. The video time code was exported down to a thousandth of a second, and turn split times were calculated in a spreadsheet (Excel 2016, Microsoft Corporation, Redmond, DC, USA). Turn split times were defined as IN-5m (last 5 m before wall contact), OUT-5m (initial 5 m following wall contact), OUT-5–10 m (from 5 m to 10 m following wall contact), and OUT-10m (initial 10 m following wall contact). Previous studies reported breakout distances of 7.76 ± 1.88 m to 5.48 ± 0.87 m in 100 m and 1500 m freestyle races, respectively [21,25]. Therefore, total turn times were determined as Total-10 (from 5 m before to 5 m following wall contact) and Total-15 (from 5 m before to 10 m following wall contact). Inter-rater reliability of turn times was reported previously with an intra-class correlation coefficient of 0.99 for males [16] and 1.00 for female swimmers [24].

### 2.3. Statistical Analysis

Descriptive statistics for the eight finalists and the eight best but non-qualified swimmers from the heats were expressed as mean ± standard deviation (SD). Shapiro–Wilk and Levene tests confirmed the assumptions of normality and homoscedasticity, respectively. All the analyses were conducted separately for both sexes [26]. CVs for each turn section were calculated according to Equation (1). As performed previously [5,6,27], a linear mixed model was applied to estimate means (fixed effects) with inter-individual CVs to compare performances of all swimmers for each of the turns separately and with intra-individual CVs including all turns of each swimmer across the race as random effects (modelled as variances). Due to the different numbers of turns, i.e., 3, 7, 15, 31, and 59, in 100 m, 200 m, 400 m, 800 m, and 1500 m races, respectively, all turns of each event were used to determine inter- and intra-individual variation in turn performances. The fixed main effects were as follows: level of performance (e.g., heats (n = 8; the best non-qualified) and finalists (n = 8)), turn number (e.g., the first, second, and third turn of a 100 m race), and turn parameter (e.g., IN-5m, OUT-5m, OUT-5-10m, OUT-10m, Total-10 and Total-15 time). To establish whether the intra-individual CV affects performance positively or negatively, the relative change in performance (∆%) was obtained using Equation (2). Subsequently, interactions between CVs, ∆%, and mean values were analysed using a two-way ANOVA (level of performance (i.e., finalists and heats) × distance (i.e., 100, 200, 400, 800-1500 m)) with Bonferroni post hoc pairwise comparisons. The effect size was expressed as *η*^2^: 0 < *η*^2^ ≤ 0.04 no effect, 0.04 < *η*^2^ ≤ 0.25 minimum effect, 0.25 < *η*^2^ ≤ 0.64 moderate effect, and 0.64 < *η*^2^ strong effect [28]. All statistical procedures were performed using SPSS 23.0 (IBM, Chicago, IL, USA), and the level of significance was set at *p* < 0.05.

(1) $$\mathrm{CV}\;=\frac{\mathrm{Standard}\;\mathrm{deviation}\;\left(e.g.,\;\mathrm{Turn}\;1\;\mathrm{and}\;\mathrm{Turn}\;2\right)}{\mathrm{Mean}\;\left(e.g.,\;\mathrm{Turn}\;1\;\mathrm{and}\;\mathrm{Turn}\;2\right)}\times 100$$

(2) $$\Delta \%\;=\frac{\mathrm{Turn}\;2\;\mathrm{performance}-\mathrm{Turn}\;1\;\mathrm{performance}\;}{\mathrm{Turn}\;1\;\mathrm{performance}}\times 100$$

## 3. Results

The results of the linear mixed-effects model analysis, intra-individual CVs, and ∆% throughout the race are presented in Table 1 for both performance levels, i.e., heat vs. final, across all race distances. Regardless of performance level, CVs and ∆% for IN-5m, OUT-5-10m, OUT-10m, Total-10, and Total-15 time showed significant variation for most races, which caused deterioration in turn performance as the race progressed. There was no significant variation in OUT-5m CV and ∆% as the race progressed (Table 1).

ANOVA testing revealed significant differences in mean turn section times, CVs, and ∆% between performance level and race distance (Table 2). In general, finalists showed faster turn section times compared to the swimmers from the heats. Short-distance races, i.e., 100 and 200 m, displayed faster turn section times than middle- and long-distance races, i.e., 400 to 800/1500 m. Additionally, finalists showed lower CVs and ∆% in all variables except for OUT-5m (men and women) and OUT-5-10m (men only), compared to swimmers from the heats. Similarly, long-distance events, i.e., 800/1500 m, showed lower mean CVs and higher mean ∆% (0.69% and 1.93%) compared to short-distance, i.e., 100 m, events (0.93% and 1.39%, respectively). However, there were no significant differences in OUT-5m with similar CVs and ∆% across the race distances. There were no significant interactions between level and distance, except for IN-5m mean values (men only) and Total-10 ∆% (women only).

**Figure 1:**
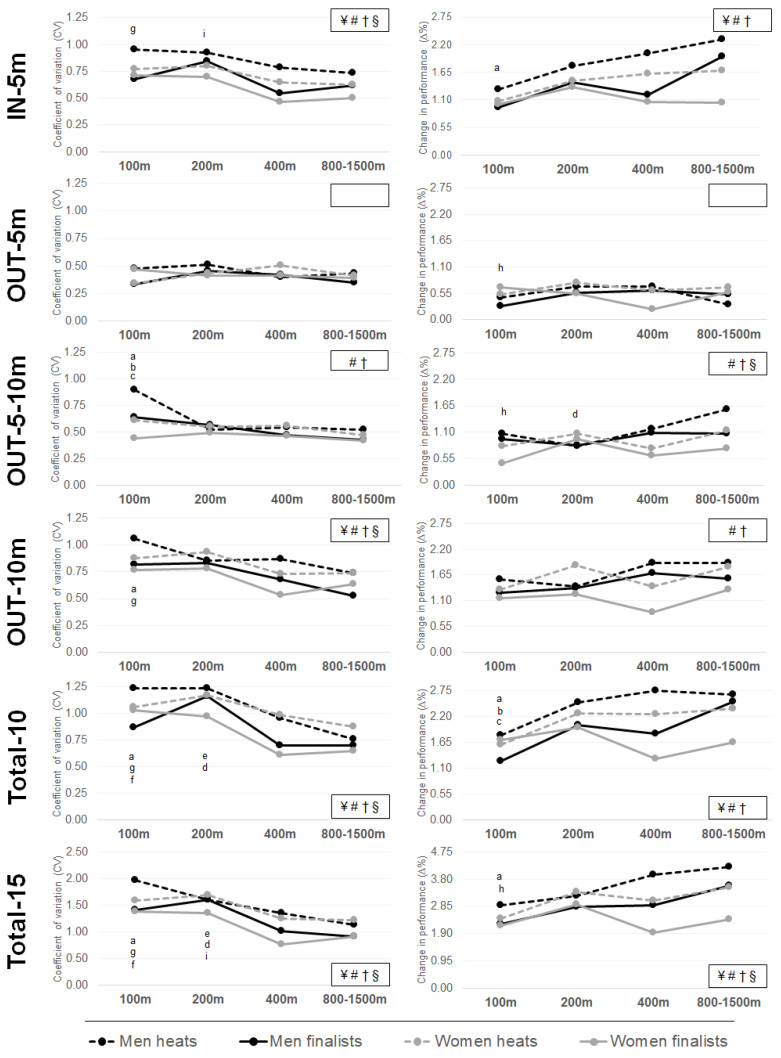
Coefficients of variation and relative changes in performance with ANOVA main effects corre-sponding to performance level and race distance for men and women. A significant main effect for: ¥ performance levels in men; # race distances in men; † performance levels in women; § race distances in women. **Significant post-hoc effect in men:** (a) between 100 and 1500 m races; (b) between 100 and 400 m races; (c) between 100 and 200 m races; (d) between 200 and 1500 m races; (e) between 200 and 400 m races; **the significant difference in women:** (f) between 100 and 800 m races; (g) between 100 and 400 m races; (h) between 100 and 200 m races; (i) between 200 and 800 m races.

All differences, specific CVs and ∆% are displayed for performance level and race distance for both sexes in Figure 1. *Post-hoc* comparisons of mean section times showed differences in all variables and both sexes between all race distances (*p* < 0.03), except for OUT-5m between 400 and 800 m in women (*p* > 0.05). Regarding intra-individual CVs in men, Bonferroni comparisons showed differences between 100 and 1500 m for OUT-10m, OUT-5-10m, Total-10, and Total-15 (*p* = 0.025; *p* = 0.001; *p* = 0.019; *p* = 0.001, respectively). Additionally, differences in OUT-5-10m between 100 and 200 m (*p* = 0.009), as well as 100 and 400 m (*p* = 0.002) were significant. Significant differences in Total-10 and Total-15 were displayed between 200 and 400 m (*p* = 0.006; *p* = 0.001, respectively) and 200 and 1500 m (*p* < 0.001; *p* = 0.002, respectively).

Bonferroni comparisons of intra-individual CVs in women showed differences in CVs between 100 and 400 m (*p* = 0.024; *p* = 0.030; *p* = 0.031; *p* = 0.001, respectively) for IN-5m, OUT-10m, Total-10, and Total-15. There were also differences between 200 and 400 m for IN-5m, OUT-10m, and Total-15 section (*p* = 0.018; *p* = 0.007; *p* = 0.001, respectively). The IN-5m and Total-15 section times showed differences between 200 and 800 m (*p* = 0.022; *p* = 0.001, respectively), as well as Total-10 and Total-15 showed differences between 100 and 800 m (*p* = 0.009; *p* = 0.003, respectively).

In men, Bonferroni comparisons for ∆% showed differences between 100 and 1500 m for IN-5m, Total-10, and Total-15 (*p* = 0.001; *p* = 0.001, *p* = 0.002, respectively). Additionally, there were differences in Total-10 between 100 and 200 m (*p* = 0.021), as well as 100 and 400 m (*p* = 0.015). Furthermore, there were significant differences in OUT-5-10m between 200 and 1500 m (*p* = 0.040). In women, Bonferroni comparisons for ∆% showed differences between 100 and 200 m for OUT-5m, OUT-5-10m, and Total-15 (*p* = 0.041, *p* = 0.047; *p* = 0.009, respectively).

## 4. Discussion

The study aimed to determine the effect of variations in short-course turn performances and investigate whether variations in turn performances differ between turn sections, race distances, and performance levels, i.e., finalists vs. non-qualified swimmers. The present study showed faster turn section times for finalists and short-distance races, i.e., 100 and 200 m, compared to non-qualified swimmers from heats and middle- and long-distance races, i.e., 400 to 800/1500 m. Except for OUT-5m, finalists showed lower CVs and ∆% for all turn section times than non-qualified swimmers. Compared to short-distance races, CVs were lower and ∆% higher in long-distance races. However, there were no significant differences in OUT-5m, with similar CV and ∆% between performance levels and distances. Turn section times increased throughout the race regardless of distance and performance level. CVs and ∆% increased when approaching the wall but not for wall exit parameters.

In short-course races, the contribution of acyclic phases, i.e., start and turns, to the total race time is widely known [16,29,30]. For instance, turn times contributed 32.92 ± 1.09% to total race time in long-course 200 m races [13] compared to 50.84 ± 0.28% in 200 m short-course races [16]. Recent research studies investigated the effect of various turn sections on race time and suggested that faster IN-5m times would increase turn performance [25,31]. Specifically, greater impulses during push-off should be achieved [32,33] by adjusting the distance between the swimmer and the pool wall [10]. In line with these previous suggestions, the present study showed a higher CV and ∆% for IN-5m compared to the wall exit sections (OUT-5m and OUT-5-10m), which showed no significant variation across all race distances. Furthermore, while there was an apparent difference between performance levels for IN-5m, statistical analysis revealed no differences for OUT-5m and OUT-5-10m.

Greater variation in the wall approach has also been found by previous research studies in long-course 200 m, 800 m, and 1500 m races [13,14,21], demonstrated by higher CVs for IN-5m (200 m: 5.10%; 800 m: 1.87%; 1500 m: 2.83%) than OUT-15m (200 m: 1.94%; 800 m 0.87%; 1500 m: 1.41%). Thus, the effect of the clean swimming section on IN-5m and the exact timing required to initiate rotation when approaching the pool wall may make this section more susceptible to fatigue as the race progresses [13,34]. This is supported by the larger effect of the IN-5m section on race time compared to the OUT-5m shown in the present data. Moreover, intra-individual comparison of turns showed progressively slower IN-5m times throughout the race, particularly for non-qualified swimmers, while OUT-5m had more constant section times. Therefore, the timing of the wall approach seems a more important determining factor for race time than wall exit parameters.

Some studies investigating key performance indicators of turn performance found velocity during the underwater phase more relevant than underwater time or distance [30,35]. Swimmers attain the highest velocities directly following push-off from the pool wall [34,36]. As the push-off from the wall ends with the head positioned at ~1.7–2.0 m, the majority of the OUT-5m section is covered by gliding in a streamlined position [36,37]. Previous studies showed little difference in the streamlined abilities between individuals [38]. However, in addition to the pure impulse during push-off, the angle at take-off and body position during the underwater phase may substantially affect turn times due to drag forces underwater [34,36,39]. Therefore, the swimmers’ ability to maintain the force from their push-off from the wall and/or to keep the correct body alignment throughout the race may be less affected by fatigue during the race and explain the lower performance variation during the wall exit section times, i.e., OUT-5m.

In contrast to the fairly consistent OUT-5m section times, OUT-5-10m performance significantly varied as the race progressed (Table 1). Increasing the gliding distance to more than 6 m after the pool wall could result in a significant loss of velocity due to the drag forces acting on the swimmer’s body [40]. Drag forces largely depend on the body’s shape and size, i.e., the general constraints of body curvatures around the head, shoulders and lumbar region [41,42]. Sex-specific anthropometric characteristics provide males with the advantage of a more cone-like body shape [43,44]. The larger hip circumference of female swimmers reduces gliding efficiency due to a less advantageous ratio between the torso and hip diameters [43,44]. However, posture and limb alignment contribute to the gliding abilities [37,42] in addition to the given anthropometric characteristics and can be altered and trained by swimmers [37]. Therefore, the gliding phase should be individualized and maintained as long as its speed is higher than the underwater kicking velocity [35,45]. The number of underwater kicks depends on the distance covered underwater, decreasing as race distances increase [21,25,46]. However, as the mean breakout distances are 7.76 ± 1.88 m to 5.48 ± 0.87 m for 100 to 1500 m freestyle races [21,25], transition to clean swimming occurs during the OUT-5-10m. The technically complex transition from the underwater to clean swimming phase requires exact timing between the initiation of flutter kicks and the first arm stroke when approaching the water surface [31,34]. This complexity may explain the larger variation in performance compared to the OUT-5m section and why finalists are better at maintaining performance throughout the race compared to non-qualified swimmers.

Differences in turn section times, i.e., IN-5m, OUT-5m, and OUT-5-10m, depended on performance level and race distance, or both, with the exception of OUT-5m (Table 2; Figure 1). As such, male and female finalists showed more consistent turn performances in all races with lower CVs than non-qualified swimmers. Turn performance variation decreased the longer the race distance. These findings align with previous studies showing low CVs between lap times in long-distance events, as consistency and even pacing are key success factors for these events [7,21]. However, it is worth noting that in some cases, e.g., for male finalists, CV for Total-15 and Total-10 increased from 100 m to 200 m before decreasing for long-distance races, thus forming an inverted U-shape pattern (refer to Figure 1). One explanation for the larger CV in 200 m events may be the effect of waves from neighboring lanes on swimmers’ front-end and back-end speed pacing [2]. Therefore, swimmers aim to reduce the effect of waves produced by the two competitors directly next to them by starting with a fast first lap into the race, i.e., high front-end speed. In contrast, the more sprint-like all-out effort of 100 m events may explain the more even pacing pattern, hence the lower CV [6,7].

The present analysis was conducted without knowledge about specific race strategies applied by the swimmers. As individual race strategies may influence turn phases, i.e., by increasing the underwater phase to reduce wave drag [47,48,49], previous studies have proposed using individualized distance, rather than fixed-distance measurements, to investigate inter-individual turn strategies [35]. However, fixed-distance measurements are still commonly used for regular assessment of the training process using simple tools, such as stopwatches, and for obtaining comparative data for race analyses during competitions [11]. Furthermore, fixed-distance measurements, i.e., OUT-5 and OUT-5-10, that include crucial parts of the turn (gliding, underwater, and transition phase) are influenced by turn velocity, which is the most relevant indicator of turn performance [30,35]. Thus, higher turn velocities resulting in shorter OUT-5 and OUT-5-10 times would serve the purpose of any race strategy.

While the present study aimed to compare fast (finalists) and slow (non-qualified) swimmers, ANOVA comparisons were limited to a low number of subjects (n = 8). Future studies could use regression analyses to compare effects across a larger sample size. Further limitations are the missing breakout distances, which provide key performance indicators for turn performances [50]. While only freestyle races were analyzed, future studies should investigate variation in turn performances in other swimming strokes, including a different turn type, i.e., open turns in butterfly and breaststroke. As the first and last lap are typically faster than the midsection [7,13,21], future studies should investigate whether the progressive decline in turn performances shown throughout races in the present study still exists when the first and last laps are excluded.

## 5. Conclusions

The wall exit variable OUT-5m showed low-performance variations across all race distances and performance levels. Due to different biomechanical constraints, i.e., wall thrust, streamline position, and underwater kicking, these turn sections seemed less affected by fatigue throughout the races. As finalists showed superior turn performances, non-qualified swimmers should aim to match the faster times of finalists in all turn sections. The largest intra-individual variation and differences between performance levels were found for IN-5m, which involves the timing of the wall approach and rotation of the body. Therefore, both finalists and non-qualified swimmers should pay particular attention to maintaining high velocities in the first section of the turn when approaching the wall as the race progresses.

From a practical perspective, coaches should adjust the wall approach strategies of their swimmers (IN-5m) according to individual technical and biological characteristics, i.e., sex, body height, race pace, and stroke length. In particular, timing and distance to the pool wall when initiating body rotation for the flip turn requires practice at swimming velocities specific to the race pace. Although lower variations were found in the wall exit phase, explosive push-off from the pool wall, streamline abilities during the gliding phase, and underwater undulating kicking affect turn performance and require particular attention during practice [34,51,52].

## Figures and Tables

**Table 1:** Intra-individual coefficients of variation (CVs) and relative changes in performance (∆%) compared with the linear mixed-effect model analysis. CV and ∆% were determined based on 3, 7, 15, 31, and 59 turns for the 100 m, 200 m, 400 m, 800 m, and 1500 m events, respectively. The eight finalists were compared with the eight fastest but non-qualified swimmers from the heats for each event.

Men			100 m			200 m			400 m			1500 m	
		CV	*p*-Value	∆%	CV	*p*-Value	∆%	CV	*p*-Value	∆%	CV	*p*-Value	∆%
IN-5m	Heat	0.95%	<0.001	1.32	0.92%	<0.001	1.78	0.79%	<0.001	2.03	0.74%	<0.001	2.31
	Final	0.67%	<0.001	0.95	0.85%	<0.001	1.44	0.54%	<0.001	1.18	0.62%	<0.001	1.98
OUT-5m	Heat	0.47%	0.142	0.46	0.52%	<0.001	0.70	0.40%	<0.001	0.70	0.43%	1.0	0.32
	Final	0.34%	0.211	0.28	0.44%	<0.001	0.55	0.42%	<0.001	0.60	0.34%	0.379	0.54
OUT-5-10m	Heat	0.90%	<0.001	1.08	0.52%	<0.001	0.81	0.55%	<0.001	1.18	0.52%	<0.001	1.57
	Final	0.64%	<0.001	0.96	0.57%	<0.001	0.83	0.47%	<0.001	1.09	0.43%	<0.001	1.08
OUT-10m	Heat	1.06%	<0.001	1.56	0.85%	<0.001	1.41	0.87%	<0.001	1.91	0.74%	<0.001	1.91
	Final	0.80%	<0.001	1.26	0.82%	<0.001	1.36	0.67%	<0.001	1.68	0.54%	<0.001	1.58
Total-10	Heat	1.23%	<0.001	1.80	1.23%	<0.001	2.49	0.96%	<0.001	2.74	0.75%	<0.001	2.66
	Final	0.83%	<0.001	1.60	1.16%	<0.001	2.01	0.70%	<0.001	1.83	0.69%	<0.001	2.52
Total-15	Heat	1.97%	<0.001	2.89	1.60%	<0.001	3.21	1.35%	<0.001	3.96	1.14%	<0.001	4.24
	Final	1.32%	<0.001	2.07	1.60%	<0.001	2.83	1.02%	<0.001	2.88	0.92%	<0.001	3.56
Women			100 m			200 m			400 m			800 m	
		CV	*p*-value	∆%	CV	*p*-value	∆%	CV	*p*-value	∆%	CV	*p*-value	∆%
IN-5m	Heat	0.77%	<0.001	1.07	0.80%	<0.001	1.48	0.65%	<0.001	1.63	0.62%	<0.001	1.68
	Final	0.71%	<0.001	1.01	0.69%	<0.001	1.35	0.46%	<0.001	1.06	0.50%	<0.001	1.05
OUT-5m	Heat	0.34%	<0.001	0.52	0.44%	<0.001	0.78	0.50%	0.001	0.61	0.41%	<0.001	0.68
	Final	0.46%	<0.001	0.67	0.41%	0.236	0.55	0.41%	0.807	0.23	0.39%	0.123	0.58
OUT-5-10m	Heat	0.61%	<0.001	0.81	0.55%	<0.001	1.08	0.56%	<0.001	0.77	0.47%	<0.001	1.14
	Final	0.45%	0.002	0.47	0.49%	<0.001	0.96	0.46%	0.228	0.53	0.42%	<0.001	0.77
OUT-10m	Heat	0.88%	<0.001	1.34	0.93%	<0.001	1.86	0.73%	<0.001	1.41	0.73%	<0.001	1.82
	Final	0.76%	<0.001	1.14	0.77%	<0.001	1.24	0.53%	0.030	0.85	0.64%	<0.001	1.34
Total-10	Heat	1.06%	<0.001	1.60	1.17%	<0.001	2.26	0.98%	<0.001	2.25	0.88%	<0.001	2.38
	Final	1.03%	<0.001	1.70	0.97%	<0.001	1.97	0.61%	<0.001	1.30	0.64%	<0.001	1.64
Total-15	Heat	1.58%	<0.001	2.41	1.68%	<0.001	3.35	1.25%	<0.001	3.04	1.22%	<0.001	3.51
	Final	1.38%	<0.001	2.17	1.36%	<0.001	2.92	0.77%	<0.001	1.92	0.92%	<0.001	2.40

**Table 2:** Comparison of means, coefficients of variation (CVs), and relative changes in performance (∆%) by a 2-way ANOVA: performance level (heat vs. final) × race distance (100 m vs. 200 m vs. 400 m vs. 800/1500 m) and corresponding effect size (*η*^2^). The eight finalists were compared with the eight fastest but non-qualified swimmers from the heats for each event.

Men		IN-5m		OUT-5m		OUT-5-10m		OUT-10m		Total-10		Total-15	
		*p*-Value	*η* ^2^	*p*-Value	*η* ^2^	*p*-Value	*η* ^2^	*p*-Value	*η* ^2^	*p*-Value	*η* ^2^	*p*-Value	*η* ^2^
Mean	Level	<0.001	0.47	<0.001	0.22	<0.001	0.25	<0.001	0.35	<0.001	0.58	<0.001	0.54
	Distance	<0.001	0.94	<0.001	0.61	<0.001	0.93	<0.001	0.90	<0.001	0.94	<0.001	0.95
	Level × Dist	0.005	0.20	0.429	0.05	0.436	0.04	0.641	0.03	0.229	0.07	0.196	0.08
CV	Level	0.007	0.12	0.166	0.03	0.054	0.06	0.025	0.09	0.014	0.10	0.016	0.10
	Distance	0.048	0.13	0.492	0.04	<0.001	0.29	0.032	0.14	<0.001	0.30	<0.001	0.31
	Level × Dist	0.625	0.03	0.678	0.03	0.163	0.08	0.696	0.03	0.393	0.05	0.366	0.06
∆%	Level	0.001	0.17	0.694	0.00	0.189	0.03	0.371	0.01	0.003	0.14	0.006	0.13
	Distance	<0.001	0.33	0.233	0.07	0.050	0.13	0.070	0.12	0.001	0.27	0.002	0.23
	Level × Dist	0.502	0.04	0.643	0.03	0.510	0.04	0.815	0.02	0.467	0.04	0.795	0.02
Women		IN-5m		OUT-5m		OUT-5-10m		OUT-10m		Total-10		Total-15	
		*p*-value	*η* ^2^	*p*-value	*η^2^*	*p*-value	*η* ^2^	*p*-value	*η* ^2^	*p*-value	*η* ^2^	*p*-value	*η* ^2^
Mean	Level	<0.001	0.53	<0.001	0.40	<0.001	0.60	<0.001	0.72	<0.001	0.77	<0.001	0.85
	Distance	<0.001	0.91	<0.001	0.67	<0.001	0.94	<0.001	0.94	<0.001	0.95	<0.001	0.97
	Level × Dist	0.511	0.04	0.914	0.01	0.107	0.10	0.374	0.05	0.463	0.04	0.059	0.12
CV	Level	0.012	0.11	0.949	0.00	0.015	0.10	0.004	0.14	0.002	0.16	<0.001	0.24
	Distance	0.001	0.25	0.811	0.02	0.355	0.05	0.002	0.23	0.008	0.19	<0.001	0.38
	Level × Dist	0.764	0.02	0.344	0.06	0.693	0.02	0.868	0.01	0.254	0.07	0.658	0.03
∆%	Level	0.002	0.16	0.249	0.02	0.017	0.09	0.001	0.17	0.001	0.18	<0.001	0.23
	Distance	0.057	0.12	0.013	0.17	0.018	0.16	0.051	0.13	0.069	0.12	0.004	0.21
	Level × Dist	0.126	0.10	0.261	0.07	0.721	0.02	0.675	0.03	0.034	0.14	0.184	0.08

## Data Availability

Data of the present investigation are available on request by the corresponding author.
